# Detection of Chromosomal Aneuploidy Using Exome Sequencing

**DOI:** 10.3390/genes16090992

**Published:** 2025-08-23

**Authors:** Mohamed H. Al-Hamed, Sateesh Maddirevula, Nabil Moghrabi, Mohammed A. Aldahmesh, Abdullah H. Alfalah, Ebtissal Khouj, Norah Altuwaijri, Midrar Alhossiny, Faiqa Imtiaz, Ahmed Alfares

**Affiliations:** 1 Precision Medicine Laboratory Department, Genomic Medicine Center of Excellence (GMCoE), King Faisal Specialist Hospital and Research Center, Riyadh 11211, Saudi Arabia; hamed@kfshrc.edu.sa (M.H.A.-H.); msateesh@kfshrc.edu.sa (S.M.); nmoghrabi@kfshrc.edu.sa (N.M.); maldahmesh@kfshrc.edu.sa (M.A.A.); aalfalah@kfshrc.edu.sa (A.H.A.); ekhouj@kfshrc.edu.sa (E.K.); naltuwaijri@kfshrc.edu.sa (N.A.); malhossiny@kfshrc.edu.sa (M.A.); fahmad@kfshrc.edu.sa (F.I.); 2 College of Medicine, Alfaisal University, Riyadh 11533, Saudi Arabia

**Keywords:** aneuploidy, chromosome, exome sequencing (ES), trisomy, copy number variation (CNV)

## Abstract

**Background**: Chromosomal aneuploidy, characterized by an abnormal number of chromosomes, represents a significant cause of genetic disorders. While karyotyping and chromosomal microarray analysis (CMA) are established diagnostic approaches, they are limited by cost and extended turnaround times. Advances in exome sequencing (ES) bioinformatics enable detection of chromosomal aneuploidy alongside single-nucleotide variant analysis. This study explores the utility of clinical ES for the detection of aneuploidies. **Method**: We analyzed exome sequencing data (2023–2024) from samples positive for Trisomy 21 (*n* = 27), Trisomy 18 (*n* = 4), Turner syndrome (*n* = 3), and Klinefelter syndrome (*n* = 2) from our clinical ES cohort (*n* = 10,000). **Results**: The results obtained were concordant with copy number variants (CNVs) identified by clinical testing. **Conclusion**: In conclusion, our findings suggest that exome sequencing offers a rapid and viable approach for the detection of chromosomal aneuploidy, potentially reducing turnaround time and associated costs.

## 1. Introduction

Chromosomal aneuploidies are a significant contributor to genetic disorders, developmental abnormalities, and pregnancy loss. Trisomy 21 (Down syndrome) and Trisomy 18 (Edwards syndrome) are among the most common aneuploidies observed in live births. Trisomy 21 occurs in 1 in every 691 births. Trisomy 18 occurs in approximately 1 in 6000–8000 live births [1]. 

Fluorescence in situ hybridization (FISH) and chromosomal microarray analysis (CMA) are some of the traditional ways to detect chromosomal aneuploidies [2]. While these methods are effective, they can be time-consuming and may have limitations in resolution [2]. Cytogenetic techniques, such as karyotyping and FISH, are commonly used but have limitations in accurately and comprehensively detecting chromosomal aneuploidies [3]. Both techniques are limited in resolution: karyotyping typically detects larger than 5–10 mb, and FISH, although more sensitive, is restricted to specific probe-targeted regions. Consequently, both may miss microdeletions, microduplications, or cryptic structural rearrangements. Karyotyping is time-consuming due to cell culture and metaphase chromosome preparation, which can delay results by several days. It is also dependent on successful cell division, which may not always be feasible in clinical samples. Both techniques have limited sensitivity in detecting low-level mosaicism, often missing anomalies present in less than 10–15% of cells [4]. FISH, while faster and usable on interphase nuclei, requires prior knowledge of the chromosomal abnormality to design specific probes [5]. This targeted approach prevents genome-wide assessment and limits its utility in cases of unknown or complex abnormalities. Additionally, balanced structural rearrangements or copy-number events outside the probe regions cannot be detected [6].

Exome sequencing (ES) is a next-generation sequencing technology that primarily targets the protein-coding regions of the genome, known as exons, but can also capture adjacent non-coding regions. It has emerged as a powerful tool for identifying genetic variations associated with various diseases [7]. Recent investigations have explored the application of exome sequencing in identifying chromosomal aneuploidy, particularly in the diagnostic evaluation of neoplastic conditions [8]. However, the utility of exome sequencing in detecting chromosomal aneuploidies outside cancer diagnosis and genetic counseling requires further investigation [9,10,11]. This study investigated the effectiveness of exome sequencing in detecting chromosomal aneuploidy. 

## 2. Methods

### 2.1. Study Design and Sample Size

This is a retrospective analysis of the exome testing results conducted at the Precision Medicine Laboratory, Genomic Medicine Center of Excellence (GMCoE), King Faisal Specialist Hospital & Research Centre, Riyadh, Saudi Arabia, from 2023–2024. The objective is to identify chromosomal aneuploidy cases that were detected during routine clinical testing (SNParray) in our accredited laboratory by the College of American Pathologists (CAP). These patients were identified as part of their routine medical care in a clinical setting. The samples used for this study were collected as part of standard clinical care and subsequently underwent exome testing. In some cases, exome testing is performed on these samples without prior knowledge or suspicion of the patients’ chromosomal aneuploidy diagnosis. 

### 2.2. Exome Sequencing

#### 2.2.1. Sample Collection and Processing 

Samples were collected according to standard clinical laboratory procedures. DNA was extracted from the samples using commercially available kits, following the manufacturer’s instructions. Exome libraries were prepared using Illumina DNA Prep with Exome 2.5 Enrichment kits, following the manufacturer’s protocol (Illumina, San Diego, CA, USA). DNA sequencing was performed using either an Illumina NovaSeq 6000 or Illumina NovaSeq X Plus. 

#### 2.2.2. Bioinformatic Analysis

The Illumina DRAGEN Bio-IT Platform, version DRAGEN 4.0.3, was used to analyze the samples. The platform converted raw signals from the Illumina NovaSeq 6000 or Nova X Plus sequencer into FASTQ format nucleotide sequences. These sequences were then aligned with the human reference genome GRCh37 to generate BAM files. The Illumina Exome 2.5 Panel HG19 bed file targeted specific genomic regions for variant detection. Variant calling was performed to identify SNVs, insertions, deletions, and CNVs, and the variants were called in VCF format. ANNOVAR (version 2019) and the local API database, along with external databases such as OMIM, ClinVar, HGMD, and Varsome were used for variant annotation, including SNV and CNV. Quality control measures were implemented at the base calling and alignment levels, including a minimum total base pair threshold of 1 Gbp and a target coverage of at least 90% at 10× depth of exome. A variant filtering algorithm was used to distinguish clinically significant variations from benign ones. All variations identified by DRAGEN at the base pair level and CNV were documented.

The Illumina DRAGEN CNV standard pipeline was used to find aneuploidy by comparing the read depth across each chromosome and comparing it to the expected diploid copy number based on a group of normal samples and batch normalization. Additionally, CNV analysis was performed to detect extra (trisomy) or missing (monosomy) chromosomes. CNV analysis looks at the read depth of a chromosome in a sample and compares it to a reference genome. This helps find places where the number of copies differs, such as duplications or deletions. The ClinGen CNV Pathogenicity Calculator (https://cnvcalc.clinicalgenome.org/redmine/projects/cnvcalc/cnv_calculator/selector 10 June 2025) was used to classify the CNVs. Results were compared to local and international CNV databases. The study followed the guidelines set by the Clinical Genome Resource (ClinGen) and the American College of Medical Genetics and Genomics (ACMG) for interpreting and reporting constitutional copy-number variants. 

### 2.3. Methodology for DNA Sequencing in Prenatal Samples (Amniotic Fluid or CVS)

Amniotic fluid or chorionic villi are collected during pregnancy under ultrasound guidance. The fetal cells are isolated, and genomic DNA is extracted using standard protocols. DNA quality and quantity are assessed before sequencing. Bioinformatics analysis and human identifier validation are included in the protocol to ensure accurate results and avoid maternal cell contamination (MCC).

### 2.4. Preliminary Observations on Suspected Aneuploidy by Physician

A retrospective review was conducted on 36 patient records where aneuploidy was suspected following the primary physician’s evaluation. The suspected aneuploidy was noted to evaluate and guide the physician’s clinical assessment in the molecular results analysis. 

### 2.5. Results Confirmation

All ambiguous results were verified by alternative methods (e.g., karyotyping, FISH, chromosomal microarray). Cases with high clinical suspicion based on presentation were not confirmed, as the results align with the clinical phenotype.

### 2.6. Ethical Approval

This study was conducted in accordance with ethical guidelines and principles. The research protocol was reviewed and approved by the Institutional Review Board (IRB) of King Faisal Specialist Hospital and Research Centre with ethical approval number (RAC# 2241173).

## 3. Results

### 3.1. Overall Detection of Aneuploidies Using Exome Sequencing 

Between 2023 and 2024, approximately 10,000 ES tests were conducted at the Precision Medicine Laboratory for clinical testing. From these 10,000 exomes, we retrospectively analyzed cases for chromosomal aneuploidies. Chromosomal aneuploidies were identified in 36 analyzed samples. These aneuploidies included Trisomy 21 (27 cases), Trisomy 18 (4 cases), Turner syndrome (3 cases), and Klinefelter syndrome (2 cases). The results from exome sequencing were consistent with those obtained from CMA (Table 1).

In our patient group, Trisomy 21 was the most frequent finding (n = 27). Of these, 18 cases were previously known, while ES solved 7 additional Trisomy 21 cases. These new cases included 4 prenatal and 3 postnatal pediatric patients. Trisomy 18 was detected in four prenatal cases, each presenting a unique phenotype linked to Trisomy 18. Klinefelter syndrome was detected in 2 cases; the first case was a 3-year-old boy with high triglycerides, a phenotype associated with Klinefelter patients. The second case was a 16-year-old male with a learning disability and hypospadias. Both cases were tested without previous genetic testing, and the CMA analysis confirmed the 47, XXY findings. Turner syndrome (45, X0) was identified in 3 cases. Two were previously known to have monosomy X, and a newly detected prenatal case (Table 1).

### 3.2. Prenatal Exome Sequencing for Aneuploidies 

ES was used as a validated clinical test in our lab for both prenatal and postnatal cases. This testing followed the College of American Pathologists’ (CAP) guidelines. Of the total cases with aneuploidies examined, 11 (30%) were prenatal, and 25 (70%) were postnatal, highlighting the broad application of this technology in diagnosing prenatal and postnatal genetic conditions (Table 1).

### 3.3. Clinical Suspicion of Aneuploidy

The physician suspected chromosomal aneuploidy in 22 out of 36 cases (61%), including 20 cases of Trisomy 21 and two of Turner syndrome. This means that 61% of physician’s evaluations were confirmed. Physicians did not suspect 14 (39%) cases of chromosomal aneuploidy, including two (100%) cases of Klinefelter syndrome, four (100%) cases of Trisomy 18, seven (26%) cases of Trisomy 21, and one (33%) prenatal case of Turner syndrome. Five of these were postnatal, and nine were prenatal (Table 1).

### 3.4. Confirmation Status

**Figure 1:**
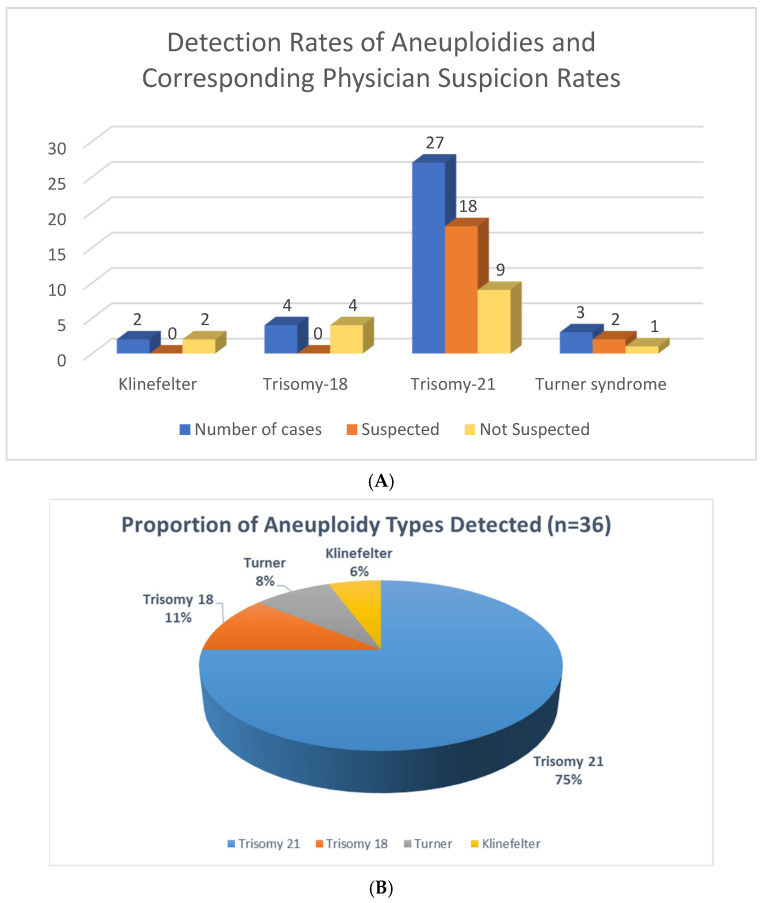
(**A**) A quantitative analysis of detected aneuploidy cases alongside the proportion of these cases that were clinically suspected by a physician. The “Detected Rate per Aneuploidy” refers to the total number of times a specific chromosomal abnormality was identified within the study cohort. The “Rate of Suspected Aneuploidy by Physician” indicates the subset of these detected cases where a physician had a pre-existing clinical suspicion of aneuploidy prior to definitive diagnosis. (**B**) Percentage of Aneuploidy Types Detected in the study.

Out of the total number of cases, 19 were verified and confirmed using alternative methods such as Chromosomal Microarray Analysis (CMA). The remaining 17 cases did not require additional confirmatory testing. Confirmation was required when prenatal imaging or screening tests were inconclusive, the clinical presentation was unclear, or the case was prenatal. However, in cases where there was a strong clinical suspicion of a specific diagnosis, such as Down syndrome or Turner syndrome, confirmation was not required (Table 1) (Figure 1).

## 4. Discussion

ES offers several advantages over traditional cytogenetic methods. It is a relatively rapid and cost-effective approach that can be performed on small amounts of DNA [12]. Exome sequencing provides more in-depth coverage of the coding region. This more targeted approach has helped to distinguish germline and somatically acquired variation by comparing normal and control or tumor samples [13]. While the initial cost of exome and genome sequencing might be higher, it can be more cost-effective in the long run, as it can detect a wider range of genetic variations compared to traditional methods such as karyotyping and CMA [14]. Next generation sequencing (NGS) demonstrates comparable or superior accuracy, turnaround time, and cost-effectiveness relative to CMA, offering substantial benefits, particularly in prenatal diagnosis [15]. Furthermore, NGS provides a comprehensive analytical approach, enabling the identification of additional genetic variations (SNVs) that may influence the patient’s phenotype [16,17]. The ability of NGS to detect mosaic cases is also a critical advantage, as other methodologies may misdiagnose these cases [18]. In addition, ES/GS demonstrates applicability for routine preimplantation genetic testing and facilitates customization of the procedure for individual patients, enabling personalized diagnostics [19]. Exome sequencing has emerged as a valuable tool for the detection of chromosomal aneuploidy across various clinical settings [20]. Although not initially designed for aneuploidy detection, advances in bioinformatics have expanded aneuploidy detection with better data coverage and read count-based approaches [21,22].

This study analyzed data from 36 samples that tested positive for aneuploidies, including 27 with Trisomy 21 and 4 with Trisomy 18, three with 45X0, and 2 with 46, XXY. These results suggest ES is an effective approach for detecting common chromosomal aneuploidies, with significant implications for prenatal and postnatal diagnosis. 

Clinical suspicion demonstrated a 61% confirmation rate for chromosomal aneuploidies, successfully identifying 20 of 27 suspected Trisomy 21, and 2 out of 3 Turner syndrome cases. However, a significant 39% (*n* = 14) of aneuploidies were not suspected clinically, encompassing all cases of Klinefelter syndrome (*n* = 2) and Trisomy 18 (*n* = 4), 26% of Trisomy 21 cases (*n* = 7), and 33% of prenatal Turner syndrome cases (*n* = 1). Notably, the missed diagnoses were observed in both postnatal (*n* = 5) and prenatal (*n* = 9) settings, highlighting the limitations of relying solely on clinical evaluation for the comprehensive detection of chromosomal aneuploidy (Figure 1). 

Several factors could explain the discrepancy between the number of suspected and confirmed cases. These include overlapping symptoms among disorders, the inherent limitations of screening test accuracy, and variability in clinical judgment. The discrepancy underscores the importance of employing advanced tools such as exome and genome sequencing in diagnostic practice, particularly in cases where clinical suspicion is low.

Exome sequencing (ES) has inherent limitations, including its inability to detect triplet repeat expansions (e.g., FMR1) and large chromosomal rearrangements, which are more accurately identified through conventional karyotyping. In addition, balanced translocations and certain cases of mosaicism may require complementary cytogenetic analyses for accurate detection. CMA remains useful for genome-wide CNV detection, especially when ES coverage is insufficient.

Compared with CMA, ES offers advantages in turnaround time and combined detection of SNVs and CNVs. However, CMA remains useful for genome-wide CNV detection, especially when ES coverage is insufficient. ES may be most beneficial for patients undergoing sequencing for other indications or in complex, multi-gene disorders.

Our findings highlight the significant potential of exome sequencing as a clinically valuable tool for prenatal chromosomal aneuploidy testing. These results support the growing body of evidence indicating that exome sequencing offers a more comprehensive and accurate approach to prenatal genetic diagnosis, particularly in cases where traditional methods may fail to detect certain genetic conditions. Furthermore, the prenatal detection of these aneuploidies facilitates more informed decision-making, earlier intervention, and improved management of affected pregnancies. Given these findings, continued development and implementation of exome sequencing in prenatal testing is recommended, especially for its ability to identify conditions that may not be clinically suspected but are critical for optimal prenatal care. Future research should focus on refining clinical guidelines and expanding the use of this technology to improve its accessibility and accuracy in prenatal diagnostics. Additionally, the ethical considerations associated with using exome and genome sequencing in prenatal diagnosis, including informed consent, data privacy, and the possibility of incidental findings, require careful attention. No incidental findings were identified in this cohort, but ethical considerations remain critical when reporting such findings. False positives and negatives are possible but mitigated by stringent QC and confirmatory testing. The relatively small number of Turner and Klinefelter cases limits statistical significance; larger studies are needed.

Clinical guidelines should integrate ES for suspected genetic syndromes, uncertain prenatal findings, and cases where broader genomic analysis is warranted. Complementary testing (karyotyping or CMA) remains essential in selected scenarios.

Further research is warranted to validate exome and genome sequencing use in a larger cohort and to investigate their potential for identifying other chromosomal abnormalities, such as deletions and translocations.

### Limitations

This study is limited by its retrospective nature and the small sample size. Further prospective studies with larger cohorts are needed to validate these findings and to explore the factors influencing the accuracy of initial suspicion.

## 5. Conclusions

In conclusion, this work demonstrates the utility of exome sequencing for detecting chromosomal aneuploidy in a cohort of 36 samples. The findings support the potential of exome sequencing as a valuable tool for aneuploidy detection, offering a more rapid, comprehensive, and potentially cost-effective single-test approach compared to traditional methodologies.

## Figures and Tables

**Table 1:** A summary of chromosomal aneuploidy cases detected using exome sequencing. The table includes information on the type of aneuploidy, associated phenotypes, whether the case was prenatal or postnatal, if the results were confirmed by another method, and whether the aneuploidy was suspected by the physician.

No	Chromosomal Aneuploidy	Phenotype	Prenatal/Postnatal	Confirmed with Different Method	Suspected by Physician
1	Klinefelter	A three-year-old boy with high triglyceridemia	Postnatal	Yes	No
2	Klinefelter	Learning disability, hypospadias	Postnatal	Yes	No
3	Trisomy-18	Abnormal fetus with strawberry-shaped head, polyhydramnios, cardiomegaly, aortic stenosis, and Trisomy 18	Prenatal	Yes	No
4	Trisomy-18	Abnormal fetus with cystic hygroma and abnormal lower limb extremities	Prenatal	No	No
5	Trisomy-18	A fetus with multiple congenital anomalies	Prenatal	No	No
6	Trisomy-18	Abnormal fetal development, including cystic hygroma and abnormal lower limb extremities	Prenatal	Yes	No
7	Trisomy-21	2-year-old boy known for cases of down syndrome with hypothyroidism, ASD and neutropenia, genetic workup for bone marrow transplant	Postnatal	Yes	Yes
8	Trisomy-21	A 2-year-old girl with Down syndrome, pancytopenia, recurrent fever, thrombocytopenia, hypothyroidism, abnormal hemoglobin, leukopenia, and splenomegaly	Postnatal	Yes	Yes
9	Trisomy-21	Known case of Down syndrome and unexplained recurrent chest infection	Postnatal	Yes	Yes
10	Trisomy-21	Down syndrome and methemoglobinemia	Postnatal	Yes	Yes
11	Trisomy-21	Anemia, neutropenia, pancytopenia, bone marrow failure and down syndrome	Postnatal	Yes	Yes
12	Trisomy-21	Two-year-old female with Trisomy 21, consort heart disease, recurrent fungal infection, severe combined immunodeficiency	Postnatal	Yes	Yes
13	Trisomy-21	A fetus at 17 weeks’ gestation with hydrops fetalis, abnormal heart, echogenic bowels	Prenatal	Yes	No
14	Trisomy-21	Abnormal fetus with severe congenital diaphragmatic hernia and dilated loops of bowel	Prenatal	Yes	No
15	Trisomy-21	A 9-year-old male diagnosed with Trisomy 21, autism spectrum disorder	Postnatal	Yes	Yes
16	Trisomy-21	One year and 8 months female with atrial septal defect, ventricular septal defect, failure to thrive, microcephaly, IUGR, sepsis	Postnatal	No	NO
17	Trisomy-21	Three years girl with hypotonia	Postnatal	Yes	No
18	Trisomy-21	Five years boy with clinical features of Down syndrome	Postnatal	No	Yes
19	Trisomy-21	A 12-year-old boy with Down syndrome, Trisomy 21, autistic spectrum disorder, congenital heart disease, and polycythemia	Postnatal	No	Yes
20	Trisomy-21	A 4-year-old male diagnosed with Abnormal pigmentation, multiple café au lait spots, speech delay, and facial dysmorphism. A known case of Down syndrome with clinical features resembling neurofibromatosis	Postnatal	No	Yes
21	Trisomy-21	Down syndrome, hypothyroidism, severe psoriasis, psoriatic arthritis, Mild macrocytosis	Postnatal	No	Yes
22	Trisomy-21	An 8-year-old boy with a known case of Down Syndrome	Postnatal	No	Yes
23	Trisomy-21	A 7-year-old girl with Down Syndrome.	Postnatal	No	Yes
24	Trisomy-21	Abnormal fetus with dilated ventriculomegaly with partial agenesis of falx cerebri and mild hydronephrosis	Prenatal	Yes	No
25	Trisomy-21	A 2-year-old male diagnosed with recurrent chest infections (pneumonia, bacteremia), developmental delay, brain atrophy, suspected Trisomy 21.	Postnatal	No	Yes
26	Trisomy-21	Suspected Trisomy 21 based on non-invasive prenatal screening	Prenatal	Yes	Yes
27	Trisomy-21	A 2-month-old female diagnosed with Atrial septal defect, hypothyroidism, hypertelorism, hypotonia, neonatal thrombocytopenia, and facial dysmorphism. Clinical features suggestive of Trisomy 21	Postnatal	No	Yes
28	Trisomy-21	A 2-year-old male diagnosed with Down syndrome, elevated liver enzymes, hepatomegaly, repeated infection (chest and chronic), chronic diarrhea, and recurrent vomiting	Postnatal	No	Yes
29	Trisomy-21	3-year-old boy with clinical features of Down syndrome	Postnatal	No	Yes
30	Trisomy-21	Abnormal fetus with thick nuchal translucency and atrial septal defect (ASD)	Prenatal	Yes	No
31	Trisomy-21	A 5-month-old male diagnosed with muscle weakness, facial dysmorphism, hypertelorism, cardiomyopathy, ventricular septal defect, failure to thrive, gross motor delay, hypotonia, aganglionic megacolon, possible Hirschsprung disease, and pneumonia	Postnatal	Yes	No
32	Trisomy-21	Abnormal fetus with cystic hygroma	Prenatal	No	Yes
33	Trisomy-21	A 7-month-old male diagnosed with Trisomy 21	Postnatal	No	Yes
34	Turner syndrome	Fetus with cystic hygroma	Prenatal	Yes	No
35	Turner syndrome	Turner syndrome, hypogonadism	Postnatal	No	Yes
36	Turner syndrome	Short stature, aortic dilatation, a webbed neck, mental retardation, autism spectrum disorder, hepatomegaly, Turner syndrome, and a large hepatic adenoma	Postnatal	No	Yes

## Data Availability

The original contributions presented in the study are included in the article, further inquiries can be directed to the corresponding author.
